# Further psychometric evaluation of the Structured Multidisciplinary Work Evaluation Tool (SMET) questionnaire: Practical implications in healthcare settings

**DOI:** 10.3233/WOR-210095

**Published:** 2022-12-13

**Authors:** Patrik Haraldsson, Bo Rolander, Dirk Jonker, Erik Strengbom, Kristina Areskoug Josefsson

**Affiliations:** a Occupational Safety and Health Care, Region Jönköping County, Jönköping, Sweden; b Department of Behavioural Science and Social Work, School of Health and Welfare, Jönköping University, Jönköping, Sweden; c Futurum – Academy for Health and Care, Region Jönköping County, Jönköping, Sweden; d Faculty of Health Studies, VID Specialized University, Sandnes, Norway; e School of Health and Welfare, Jönköping Academy for Improvement of Health and Welfare, Jönköping University, Jönköping, Sweden; f Department of Behavioural Science, Oslo Metropolitan University, Oslo, Norway

**Keywords:** Work environment, occupational health service, questionnaire, reliability, validity

## Abstract

**BACKGROUND:**

Scientific research has identified a lack of psychometrically well-tested methods for evaluation of the work environment in healthcare settings. The Structured Multidisciplinary Work Evaluation Tool (SMET) questionnaire has been evaluated and has shown good content validity, as well as intra-rater and test-retest reliability. There are, however, still unknowns regarding the psychometric properties. If the SMET questionnaire is to be used in practical occupational health service (OHS) work and scientific research in healthcare settings, further psychometric evaluation is needed.

**OBJECTIVE:**

The aim of this study was to gain further understanding of the psychometric properties of the SMET questionnaire when used in research and clinical OHS practice in healthcare settings.

**METHODS:**

The psychometric evaluation was conducted using classical test theory (Cronbach’s alpha, explorative factor analysis) and Rasch analysis (measurement targeting, category threshold order, person separation index) on data previously collected in development projects within the healthcare sector.

**RESULTS:**

The results support the use of the SMET questionnaire as a psychometrically well-tested method for evaluation of the work environment in healthcare settings. They support the use of the initial 1–10 scale since all 10 steps are used. The results also support the trichotomization procedure since the trichotomized scale captures the construct of the work environment with good measurement targeting and good category threshold order.

**CONCLUSION:**

The results of this study support the use of the SMET questionnaire as a psychometrically well-tested method for a broad multifactorial evaluation of the work environment in healthcare settings.

## 1 Introduction

Participatory organizational interventions are seen as potentially sustainable and effective approaches to improving worker safety, health, and wellbeing [1, 2]. In 2008, the European Agency for Safety and Health at Work stated that Occupational Health Service (OHS) services should be based on active participation, risk identification and interventions from a multidisciplinary/multifactorial perspective [3]. Since OHS work in Sweden relies heavily on curative services (such as rehabilitation) offered on an individual level, rather than preventive issues with a systems approach [4], this implies a need for change in the present work of OHS in Sweden. Research concerning OHS participatory and multidisciplinary interventions has shown the need for effective evaluation measurements targeting such interventions to avoid misinterpretation of outcomes [1, 2, 5].

The work environment consists of physical, environmental and organizational/social aspects that interact with each other in complex ways [6, 7], making work environment evaluations challenging.

There are several evaluation methods used for general evaluation of the work environment, used in both research and clinical practice. Examples are Borg’s RPE scale, by which employees rate perceived workload [8] and the Standardized Nordic Questionnaire by which employees rate work-related pain [9]. However, since these methods evaluate individual characteristics it is uncertain to what degree they provide information about the work environment. Quick Exposure Check is a method which evaluates physical work exposures and stress [10], but does not cover environmental or organizational/social aspects. The Copenhagen Psychosocial Questionnaire, version 3 (COPSOQ 3) is a tool for evaluation of the work environment from a psychosocial perspective, but does not include important information about physical and environmental aspects [11, 12]. All these evaluation methods have been assessed regarding their psychometric properties, but none evaluates the work environment from a broader multifactorial perspective.

A recent systematic literature review identified a lack of useful and psychometrically well-tested methods for evaluation of the work environment in healthcare settings [13], indicating an urgent need of high quality methods for evaluation of the work environment in healthcare settings.

The Structured Multidisciplinary Work Evaluation Tool (SMET) was developed based on action research from 2008 to 2014 to meet the need for effective evaluation of the work environment with a broader multifactorial approach [14, 15]. SMET consists of a questionnaire by which the employee evaluates their work environment, and an objective in-depth analysis of the workplace, performed by OHS. The SMET questionnaire identifies work environment problems from a multifactorial perspective including physically-, environmentally- and psychosocially-demanding factors. Each subfield (physical, environmental and psychosocial) also contains an item evaluating which of the subfield’s items have the highest work demands, and an open-ended item by which employees describe their work-related problems. In addition, the subfield for environmental factors is complemented with two items regarding chemical exposure [14]. The SMET questionnaire was also developed to optimize user-friendliness [14] resulting in a mean response time of 12 minutes (unpublished data), thus minimizing time-consumption at the workplace. Previous psychometric evaluations of the SMET questionnaire have shown good pragmatic and content validity [14] and good inter-rater and test-retest reliability [15]. The SMET questionnaire has been shown to reflect true physical workload in certified nursing assistants in a medical ward setting [16]. These findings support the use of the SMET questionnaire in health care settings, for multifactorial risk assessment of the work environment and follow-up/evaluation of work environment measures.

The SMET questionnaire is currently used in practical OHS work and ongoing scientific research in Sweden. However, to trust the SMET questionnaire's ability to evaluate the work environment and changes related to work environment measures, the psychometric value of the SMET questionnaire needs to be solid. Therefore further psychometric evaluations are needed to ensure high quality, validity and reliability of the SMET questionnaire. The need of further psychometric evaluations are supported by the findings in the recent systematic literature review by Maassen, et al. [13].

Further psychometric evaluations of the SMET questionnaire should include how the initial 1–10 Likert scale is used, whether the questionnaire captures the construct of the work environment as well as measurement targeting and category threshold order. The need for the trichotomization process can be questioned and needs further exploration to prove its worth. The trichotomization procedure strengthens the test-retest reliability and simplifies the result presentation, but there might be a risk of missing and misinterpreting information with this approach [17]. Further psychometric evaluations are important to increase knowledge about how to compile and interpret the results of the SMET questionnaire within a healthcare context.

Classical test theory (CTT) refers to the evaluation of scale validity, scale reliability, factor analysis etc., [18], and despite the SMET questionnaire being considered reliable and valid in previous studies with CTT [14, 15], there are still psychometric properties to explore further.

Rasch analysis is a model for psychometric evaluation of a scale, created by the Danish mathematician Georg Rasch [19] based on the probabilistic relationship between a person’s ability and item difficulty [20]. By using the Rasch model [21], further knowledge of both psychometric properties and potential changes in outcomes due to the trichotomization process can be obtained. The Rasch analysis presents information about category threshold order [20, 22, 23], measurement targeting [20], and further evaluation of internal consistency using logit values etc. [23] which makes the analysis suitable to use for questionnaire development [24].

Applying further psychometric evaluations with both the CTT and the Rasch model can add useful knowledge about the validity and reliability of the SMET questionnaire. The added knowledge will ensure high quality, validity and reliability when evaluating the work environment and the outcome of work environment measures with the SMET questionnaire in healthcare settings.

## 2 Objectives

The aim of this study was to gain further understanding of the psychometric properties of the SMET questionnaire and thereby increase the usefulness of the questionnaire in research and clinical OHS practice in healthcare settings.

*Research questions*

Are all response options in the initial 1–10 scale used?

Is the questionnaire capturing the construct of the work environment?

Is the measurement targeting in the questionnaire acceptable?

Are the category thresholds in the items naturally ordered?

Are the work environment construct, the measurement targeting and category threshold order affected by the trichotomization procedure?

## 3 Materials and methods

### 3.1 Sample

The sample group consisted of 19 work environment projects at 13 different workplaces, all in the same region in the south of Sweden. The included workplaces were mainly medical ward departments, but there was also a minor contribution from a kitchen department and a laboratory. Nurses and assistant nurses were the main professions but others, such as administrative personnel, physicians, kitchen personnel and technicians, were included. The sample was a pragmatic one, based on the departments with which the regional Occupational Safety and Health Care had been involved and which had used the SMET questionnaire between 2015 and 2018.

### 3.2 Data collection

The data consisted of 1001 answered SMET questionnaires collected from 19 work environment projects, conducted between 2015 and 2018. The SMET questionnaire was sent out to the employees using an online questionnaire system (esmaker) and was possible to answer for two to three weeks.

### 3.3 The SMET questionnaire

The SMET questionnaire contains 30 items which evaluate the workplace regarding physically- (nine items), environmentally- (eight items) and psychosocially- (13 items) demanding factors (Table 1). Twenty-two of these consist of self-reported physical (seven), environmental (four) and psychosocial workload (ten) items. Each subfield (physical, environmental and psychosocial) also contains an item that evaluates which of the previous items constitutes the highest work demand, and an open-ended item by which employees describe their work-related problems. In addition, the subfield for environmental factors is complemented with two items regarding chemical exposure [14]. Only the 22 self-estimating items were tested in the psychometric evaluation. In practical work with the SMET questionnaire, 22 of the self-estimating items (1–7, 10–13, 18–27 and 30) are answered with a 1–10 response scale (1 = no problems, 10 = major problems). The scale is then trichotomized from a 1–10 response scale to indicate work environment risk exposure [15]. The trichotomization groups’ response options 1–3, 4–7, 8–10 to three groups are presented as follows: 1–3 = low degree of problems (colored green), 4–7 = some degree of problems (colored yellow) and 8–10 = high degree of problems (colored red). The trichotomization procedure was initially used for pragmatic validity, to increase understanding of the presented outcomes when presenting the results of the SMET questionnaire to managers and staff by using the same color codes (green, yellow, red) employed by the Swedish Work Environment Authority (SWEA) [25]. Psychometric evaluation supports the use of trichotomization in the SMET questionnaire since it has shown very good test-retest reliability [15]. The SMET presentation also includes the frequencies of ratings 1 and 10 in frequencies in order to present the extremes in the explored setting. The item constituting the worst problem in each domain is presented and the open-ended items from the SMET questionnaire are analyzed with content analysis and presented as a comprehensive summary of the results [15]. Finally, the SMET presentation includes a correlation analysis of all the self-estimated items in order to evaluate how the results of the different items in the questionnaire relate to each other.

**Table 1:** All items presented with missing values in frequencies (MI), skewness (Sk) and response values in the 1-10 scale (1= no problems, 10= major problems) presented by the computer program as 0-9 in percent (%)

Response values 0–9 in %												
Item	MI	Sk	0	1	2	3	4	5	6	7	8	9
**Physically demanding work items**
1. Do you experience any problems associated with heavy lifting in your work?	–	0.09	19.7	10.5	10.5	6.3	9.0	9.5	11.5	14.1	5.4	3.7
2. Do you experience any problems associated with repetitive movements in your work?	–	0.20	13.4	11.6	14.6	10.0	11.1	9.5	9.0	12.5	5.8	2.6
3. Do you experience any problems associated with unilateral or fixed working positions in your work?	–	0.20	10.8	11.7	14.1	11.6	11.6	10.6	10.1	11.7	4.4	3.3
4. Do you experience any problems associated with uncomfortable working positions in your work?	–	–0.1	7.8	8.7	12.6	8.7	11.1	11.2	11.6	14.5	8.3	5.4
5. Do you experience any problems associated with a high work-pace in your work?	–	–0.7	2.6	3.8	4.8	7.3	8.3	7.7	14.0	18.5	14.4	18.5
6. Do you experience any problems associated with eyesight demands in your work?	–	0.33	19.4	12.1	12.0	8.8	12.9	8.1	9.1	9.7	4.0	3.9
7. Do you experience any problems associated with prolonged sitting in your work?	–	1.44*	48.3	14.6	11.1	5.7	5.2	3.4	4.3	2.9	2.0	2.5
**Environmentally demanding work items**
8. Do you experience any problems associated with high noise levels at your workplace?	–	0.70	22.1	15.9	16.6	7.6	11.0	6.8	5.3	6.7	4.0	4.0
9. Do you experience any problems associated with heat, cold, or drafts at your workplace?	–	0.45	16.8	15.3	14.4	9.3	12.5	8.2	7.8	8.4	4.2	3.2
10. Do you experience any problems associated with the lightning in your workplace?	–	1.22*	32.0	22.7	17.2	5.7	8.6	5.2	3.4	2.9	1.6	0.8
11. Do you experience any problems associated with narrow spaces in your workplace?	–	0.12	12.6	10.3	13.2	7.3	10.8	7.9	8.8	10.6	7.3	11.0
**Psychosocially demanding work items**
12. Do you experience any problems associated with work routines and the distribution of tasks at your workplace?	–	0.18	9.2	12.5	14.2	8.3	12.5	10.7	11.0	12.0	3.2	6.4
13. Do you experience any problems associated with collaboration, and feedback at your workplace?	–	0.49	11.0	17.9	15.9	10.7	12.0	9.6	6.8	8.8	3.5	3.8
14. Do you experience any problems associated with support from your boss/employer?	–	0.37	16.8	18.0	11.6	5.9	9.3	6.7	8.2	10.1	5.2	8.2
15. Do you experience any problems with responsibilities, rights and/or expectations at your workplace?	–	0.64	14.4	19.3	16.8	9.5	11.9	6.8	6.8	7.9	2.4	4.3
16. Do you experience any problems associated with your possibilities to develop in your work?	–	0.61	18.2	17.7	13.4	9.1	13.2	8.3	6.7	6.4	2.3	4.8
17. Do you experience any problems associated with unreasonable demands in your work?	–	0.25	13.7	14.4	10.6	9.6	12.4	7.9	8.8	11.3	4.8	6.4
18. Do you experience any problems associated with having control and being able to handle the psychological demands that arise?	–	0.29	13.5	14.9	12.4	9.1	11.3	10.0	10.6	9.2	3.9	5.2
19. Do you experience any problems associated with having no time to take breaks on an ordinary working day?	–	0.37	16.4	14.0	11.7	10.7	10.8	7.7	7.8	8.7	5.9	6.2
20. Do you experience any problems associated with anxiety about making serious mistakes in your work?	–	0.09	11.5	14.6	15.5	9.8	10.3	7.8	9.1	10.2	5.3	5.8
21. Do you experience any problems associated with anxiety about not having time to complete your work?	–	–0.1	8.7	10.2	10.2	7.7	10.1	9.0	11.3	13.5	7.7	11.6
22. If you think about your work satisfaction and work environment, how satisfied would you say that you are?	–	–0.3	2.3	5.7	7.3	8.1	16.0	14.1	18.3	16.6	7.3	4.3

*n* = 793. * = Highly positive skewed.

### 3.4 Data analysis

#### 3.4.1 Evaluation of response options in the 1–10 scale

Evaluation of how the respondents used response options in the 1–10 scale was presented with descriptive data frequencies (n), percent (%) and skewness (sk) for each item.

#### 3.4.2 Evaluation of the work environment construct

The construct of the work environment was evaluated with internal consistency in all 22 self-estimating items and in the domains *physical work demands* (7 items), *environmental work demands* (4 items) and *psychosocial work demands* (10 items) separately. Internal consistency was evaluated with Cronbach’s alpha in both the 1–10 and the trichotomized scale. An alpha value above 0.60 was considered good and values above 0.70 were considered optimal [26]. Additional evaluation of internal consistency was performed with the Person Separation Index (PSI) in the Rasch analysis. The PSI is an evaluation of internal consistency, similar to Cronbach’s alpha, but conducted with logit values [23]. The PSI evaluates the ability of the questionnaire respondents to separate high and low performing, with low values indicating that more items are needed [27]. A PSI value above 0.70 was considered good internal consistency [23].

Evaluation of how the work environment construct was affected by the trichotomization procedure were conducted by comparing the results of Cronbach’s alpha and PSI in the 1–10 scale and the trichotomized scale.

Further evaluation of how construct validity was affected by the trichotomization procedure was evaluated with explorative factor analysis (method principal components and varimax rotation), with extraction based on an eigenvalue greater than 1, comparing the 1–10 and the trichotomized scale. Only items with a factor loadings >0.5 were presented.

#### 3.4.3 Evaluation of measurement targeting

Measurement targeting was evaluated by central tendency and dispersion for person-item thresholds, presented in mean (m) and standard deviation (sd) for logits. The Rasch analysis generates independent estimates of the respondent and item parameters (centralized to zero) on a common logit (log-odds) scale, where the location of the items relative to the respondents can be examined. No difference (0) between person and item mean value was considered perfect targeting, and a difference greater than 1 was considered poor targeting [20].

Evaluation of how measurement targeting was affected by the trichotomization procedure was conducted by comparing the results of the person-item thresholds in the 1–10 scale and the trichotomized scale.

#### 3.4.4 Evaluation of category threshold order

Category threshold order is presented with response category probability curves. The response category probability curves show the probability that a category will be selected with regards to person ability and item difficulty [27]. Category threshold disorder reflects underused categories that might relate to an unclear description (text) of the category or more categories than used by the respondents [20].

Evaluation of how response option separation was affected by the trichotomization procedure was conducted by comparing the response category probability curves in the items of the 1–10 scale and the trichotomized scale.

#### 3.4.5 Statistics

The CTT evaluations (response options in the 1–10 scale, Cronbach’s alpha, explorative factor analysis) were analyzed in SPSS version 24 [28]. The Rasch analysis (PSI, measurement targeting, response option separation) was calculated using the RUMM 2030 (Rasch Unidimensional Measurement Model) software (standard edition) version 5.1. The Rasch analysis was conducted with a polytomous Rasch model which allowed more than two ordered categories [23].

Research suggests that sample sizes in Rasch analysis should include at least 10 observations/category [29, 30], indicating a minimum sample size of *n* = 100 in evaluation of the 1–10 scale and *n* = 30 in evaluation of the trichotomized scale. Sample sizes above *n* = 500 are considered very good in both Rasch analysis [30] and CTT (Factor analysis) [31].

### 3.5 Ethics

The data in this study was collected from practical OHS interventions in the south of Sweden. The study has not been reviewed by an institutional review board since this is not required for this type of study according to the Swedish Ethical Review Act [32]. However, the research study protocol followed the regulations in the Helsinki Declaration [33]. Data has been stored on a safe hard drive in Region Jönköping County in accordance with the General Data Protection Regulation.

## 4 Results

The study material consisted of 1001 answered questionnaires, and 793 (79%) completely answered questionnaires were used in the analyses.

### 4.1 Responses in the 1–10 scale

Two of the 22 items, items 7 and 10, were highly positively skewed. No items were moderately or highly negatively skewed. All ten response alternatives were used by the respondents. The lowest response option on all items had a mean reply proportion of 15.5 percent (CI95%  = 4.3) with the lowest proportion from 2.3 percent up to 48.3 percent. The highest response option had a reply proportion on average of 5.7 percent (CI95%  = 1.7) with the lowest reply proportion of 0.8 percent (*n* = 6) to the highest reply proportion of 18.5 percent (Table 1).

### 4.2 Work environment construct

#### 4.2.1 Internal consistency

Evaluation of the work environment construct shows that the construct of the work environment is well captured in all the 22 self-estimating items and that physically demanding work and psychosocially demanding work are captured well in their domains, both in the 1–10 scale and the trichotomized scale. These domains show very small changes in internal consistency by the trichotomization procedure. The domain of environmentally demanding work captures to a lesser degree its construct with the trichotomized scale, and might be more affected by the trichotomization procedure, as shown by the result of the PSI in this domain (Table 2).

**Table 2:** Evaluation of work environment construct with Crohnbach’s alpha and Person Separation Index (PSI) of both the 1–10 scale (1 = no problems, 10 = major problems) and the trichotomized scale

	1–10 scale	Trichotomized scale
All self-estimating items in the questionnaire (22 items)
Crohnbach’s alpha	0.89	0.87
Person Separation Index	0.90	0.86
Physically demanding work items (7 items)
Crohnbach’s alpha	0.79	0.77
Person Separation Index	0.79	0.72
Environmentally demanding work items (4 items)
Crohnbach’s alpha	0.66	0.60
Person Separation Index	0.66	0.34
Psychosocially demanding work items (10 items)
Crohnbach’s alpha	0.87	0.84
Person Separation Index	0.86	0.79

The evaluation was conducted in all 22 self-estimating items and the physical, environmental and psychosocial domains separately. *n* = 793.

#### 4.2.2 Explorative factor analyses

The factor analyses were almost identical when performed on the results of the 1–10 scale and the trichotomized scale, leading to five factors. There was a difference in cumulative percent of variance for the 1 to 10 scale of 67.9 and for the trichotomized scale of 62.4. Furthermore, the trichotomized factor analyses showed slightly lower factor values, and item 22 was present in both factor 1 and factor 3 (Table 3).

**Table 3:** Explorative factor analysis with five factors for 1 to 10 scale (1 = no problems, 10 = major problems) and trichotomized scale in factor values. *n* = 793

	1 to 10 scale					Trichotomized scale				
Percent of variance:	19.5%	17.5%	14.5%	9.2%	7.2%	17.8%	15.9%	13.6%	8.2%	6.9%
Item	1	2	3	4	5	1	2	3	4	5
Physically demanding work items
1. Do you experience any problems associated with heavy lifting in your work?			0.73					0.73
2. Do you experience any problems associated with repetitive movements in your work?			0.86					0.83
3. Do you experience any problems associated with unilateral or fixed working positions in your work?			0.81					0.79
4. Do you experience any problems associated with uncomfortable working positions in your work?			0.82					0.79
5. Do you experience any problems associated with a high work-pace in your work?	0.70					0.68
6. Do you experience any problems associated with eyesight demands in your work?					0.56					0.53
7. Do you experience any problems associated with prolonged sitting in your work?					0.87					0.86
Environmentally demanding work items
8. Do you experience any problems associated with high noise levels at your workplace?				0.52					0.48
9. Do you experience any problems associated with heat, cold, or drafts at your workplace?				0.71					0.72
10. Do you experience any problems associated with the lightning in your workplace?				0.74					0.71
11. Do you experience any problems associated with narrow spaces in your workplace?				0.56					0.51
Psychosocially demanding work items
12. Do you experience any problems associated with work routines and the distribution of tasks at your workplace?		0.72					0.68
13. Do you experience any problems associated with collaboration, and feedback at your workplace?		0.78					0.75
14. Do you experience any problems associated with support from your boss/employer?		0.78					0.77
15. Do you experience any problems with responsibilities, rights and/or expectations at your workplace?		0.74					0.73
16. Do you experience any problems associated with your possibilities to develop in your work?		0.70					0.69
17. Do you experience any problems associated with unreasonable demands in your work?	0.73					0.69
18. Do you experience any problems associated with having control and being able to handle the psychological demands that arise?	0.69					0.66
19. Do you experience any problems associated with having no time to take breaks on an ordinary working day?	0.78					0.74
20. Do you experience any problems associated with anxiety about making serious mistakes in your work?	0.76					0.73
21. Do you experience any problems associated with anxiety about not having time to complete your work?	0.84					0.83
22. If you think about your work satisfaction and work environment, how satisfied would you say that you are?		–***0.53***				–***0.49***		–***0.46***

### 4.3 Measurement targeting

**Fig. 1:**
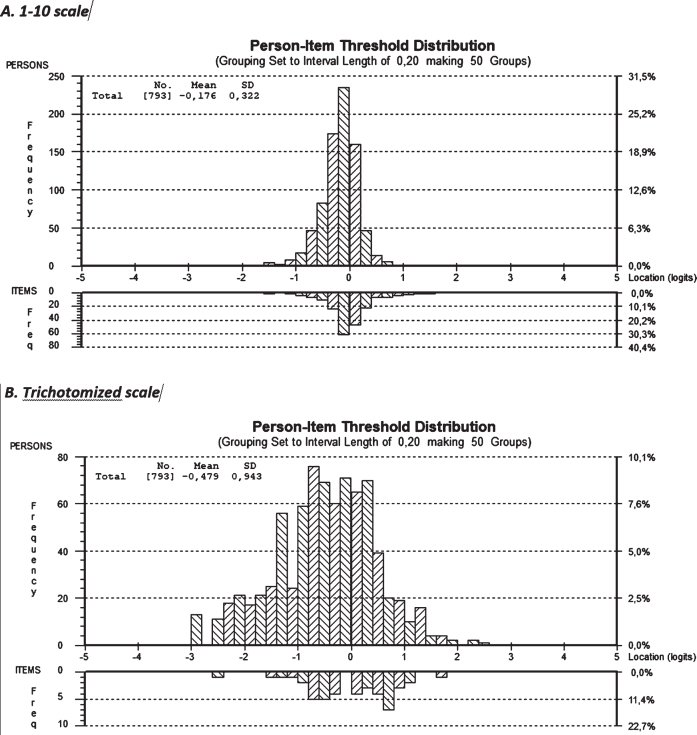
Measurement targeting, presented with person-item distribution for all 22 items in the 1–10 scale (1 = no problems, 10 = major problems) and the trichotomized scale.

Measurements showed good targeting for all 22 items in the 1 to 10 scale, as shown by the low mean and dispersion values (*m* = 0.176, SD = 0.322) of the persons estimate. At the negative end of the scale, persons were located and there were also item thresholds, but on the positive end, factor values were slightly outside, confirming the rather good targeting, which means that the items representing the sample and the 1 to 10 scale had good measurement targeting. For the trichotomized scale, the mean and dispersion values were not quite as good (*m* = 0.479, SD = 0.943) and the targeting was not as good as for the 1–10 scale, where persons on both the negative and the positive ends were outside the item scale. This indicates that the trichotomization procedure negatively affects measurement targeting (Fig. 1).

### 4.4 Category threshold order

Twenty-one of the items in the trichotomized scale showed good category threshold order, whereas only nine of the items in the 1–10 scale did so. Only item 7 in the trichotomized scale showed category threshold disorder compared with the 1 to 10 scale where 13 items showed category threshold disorder (Table 4).

**Table 4:** Category threshold order in 10-scale (1 = no problems, 10 = major problems)(left) and the trichotomized scale (right)

1 to 10 scale													Trichotomized scale					
Seq	Code	Location	Mean	CenThr 1	CenThr 2	CenThr 3	CenThr 4	CenThr 5	CenThr 6	CenThr 7	CenThr 8	CenThr 9	Seq	Code	Location	Mean	CenThr 1	CenThr 2
1	I0001	0,100928	0	0,026	–0,044	–0,177	–0,313	–0,391	–0,352	–0,136	0,318	1,069	1	I0001	–0,07008	0	–0,405	0,405
2	I0002	0,056913	0	–0,702	–0,267	–0,105	–0,109	–0,169	–0,178	–0,03	0,385	1,174	2	I0002	–0,03353	0	–0,62	0,62
3	I0003	0,019493	0	–0,764	–0,358	–0,166	–0,107	–0,099	–0,062	0,087	0,428	1,043	3	I0003	–0,04783	0	–0,811	0,811
4	I0004	–0,13431	0	–0,84	–0,344	–0,114	–0,053	–0,068	–0,062	0,06	0,392	1,029	4	I0004	–0,45752	0	–0,766	0,766
5	I0005	–0,56776	0	–0,865	–0,435	–0,165	–0,009	0,079	0,142	0,228	0,38	0,645	5	I0005	–1,65095	0	–0,941	0,941
6	I0006	0,079514	0	–0,076	–0,169	–0,216	–0,215	–0,166	–0,067	0,083	0,285	0,54	6	I0006	0,120772	0	–0,608	0,608
7	I0007	0,230296	0	0,616	0,21	–0,019	–0,117	–0,13	–0,105	–0,086	–0,119	–0,251	7	I0007	0,858432	0	0,129	–0,129
8	I0008	0,104013	0	–0,332	–0,113	–0,008	0,021	0,009	–0,005	0,015	0,106	0,305	8	I0008	0,419181	0	–0,368	0,368
9	I0009	0,084622	0	–0,43	–0,189	–0,087	–0,07	–0,082	–0,069	0,022	0,247	0,659	9	I0009	0,223975	0	–0,558	0,558
10	I0010	0,435049	0	–0,473	–0,167	–0,079	–0,117	–0,191	–0,207	–0,074	0,3	1,007	10	I0010	1,230151	0	–0,465	0,465
11	I0011	–0,13944	0	–0,321	–0,114	0,006	0,061	0,073	0,064	0,053	0,063	0,115	11	I0011	–0,29813	0	–0,342	0,342
12	I0012	–0,06623	0	–0,784	–0,376	–0,153	–0,052	–0,01	0,035	0,145	0,383	0,812	12	I0012	–0,12901	0	–0,821	0,821
13	I0013	0,049337	0	–0,955	–0,34	–0,042	0,043	0,019	–0,011	0,057	0,327	0,902	13	I0013	0,218627	0	–0,715	0,715
14	I0014	–0,01736	0	–0,337	–0,01	0,099	0,066	–0,033	–0,122	–0,126	0,033	0,43	14	I0014	0,001851	0	–0,225	0,225
15	I0015	0,087736	0	–0,854	–0,282	–0,005	0,071	0,041	–0,002	0,037	0,253	0,74	15	I0015	0,390718	0	–0,632	0,632
16	I0016	0,09231	0	–0,382	–0,259	–0,152	–0,057	0,027	0,103	0,173	0,241	0,307	16	I0016	0,415713	0	–0,748	0,748
17	I0017	–0,02428	0	–0,512	–0,231	–0,091	–0,043	–0,035	–0,017	0,063	0,255	0,611	17	I0017	–0,09417	0	–0,67	0,67
18	I0018	0,022764	0	–0,563	–0,289	–0,149	–0,093	–0,068	–0,022	0,095	0,336	0,753	18	I0018	0,080972	0	–0,808	0,808
19	I0019	–0,00761	0	–0,315	–0,159	–0,069	–0,022	0,003	0,027	0,071	0,157	0,306	19	I0019	0,017537	0	–0,545	0,545
20	I0020	–0,0358	0	–0,815	–0,243	0,023	0,081	0,03	–0,029	0,001	0,221	0,73	20	I0020	–0,00628	0	–0,556	0,556
21	I0021	–0,20927	0	–0,498	–0,222	–0,068	0,001	0,024	0,04	0,087	0,205	0,432	21	I0021	–0,57068	0	–0,602	0,602
22	I0022	–0,16091	0	–0,46	–0,318	–0,239	–0,189	–0,133	–0,037	0,133	0,411	0,832	22	I0022	–0,61976	0	–0,805	0,805

The marked items in the scales shows category threshold disorder. *n* = 793.

**Fig. 2:**
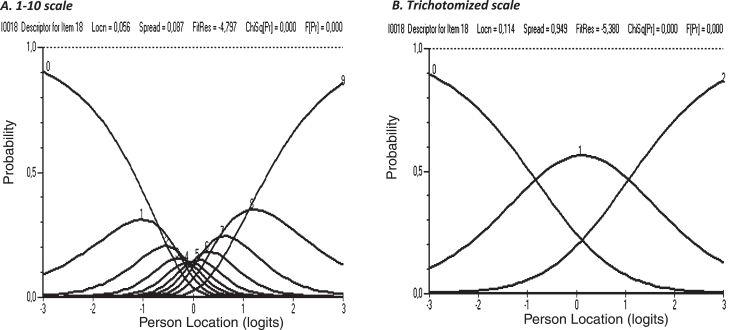
An example of clear difference in category threshold order between the 1–10 scale (1 = no problems, 10 = major problems) and the trichotomized scale (item18).

For the 1 to 10 scale it was apparent that only the external categories (1 and 10) were correctly ordered whereas the trichotomized scale showed clear category threshold order, shown with the example of item 18 (Fig. 2).

**Fig. 3:**
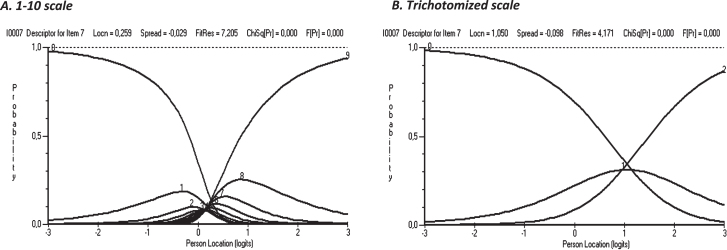
An example of category threshold disorder in both the 1–10 scale (1 = no problems, 10 = major problems) and the trichotomized scale (item 7).

However, one item, (item 7) showed category threshold disorder even when trichotomized and none of the scales had a clear category threshold order (Fig. 3).

## 5 Discussion

### 5.1 Results discussion

The results of this study support the use of the SMET questionnaire as a method with high psychometric quality, useful for a broad multifactorial evaluation of work environments in healthcare settings. This is an important finding since a recent systematic literature review identified a need of high quality methods for evaluation of the work environment in healthcare settings [13].

The results show that all 10 steps of the initial 1–10 scale were used by the respondents, supporting the use of the initial 1–10 scale. The SMET questionnaire is able to capture the construct of the work environment as well as the domains of physical and psychosocial work demands due to its good internal consistency reliability for both the 1–10 scale and the trichotomized scale. The reliability of the SMET questionnaire is therefore further strengthened, and the questionnaire can thus be confirmed to measure the construct of the work environment. These results and the results from comparing the factor analysis support the trichotomization procedure, since it had a minimal effect on the construct of the SMET questionnaire. However, the SMET questionnaire might be less able to capture the construct of environmental work demands with the trichotomized scale, indicating that more items should be added in this domain in future revisions of the questionnaire. To the best of our knowledge trichotomization is not used in other work environment methods. Scientific research on neurophysiological tests for evaluation of fitness to drive has conducted serial trichotomization. This serial trichotomization improved the sensitivity and specificity of testing [34, 35] and improved the ability to predict driving test outcome [34], supporting the use of trichotomization in scales.

The person-item threshold distribution showed very good measurement targeting with the 1–10 scale, and the result with the trichotomized scale, as expected, showed less precision. The only (to our knowledge) work environment questionnaire that has analyzed measurement targeting with Rasch is the workplace social capital domain of COPSOQ 3. The mean difference between person ability and item difficulties in this domain of COPSOQ 3 was 1.217 [36]. Comparison with workplace social capital domain of COPSOQ 3 and the fact that the trichotomized scale was well within the 0-1 range of good/acceptable targeting supports good targeting in the trichotomized scale of the SMET questionnaire.

The study results showed that category threshold order was much better with the trichotomized scale, which is also an expected finding. The trichotomization has pedagogical value when presenting results to management and staff, as OHS often present their results in red-yellow-green (high degree of problems-moderate degree of problems-low degree of problems), indicating the need for occupational health interventions [25]. The only (to our knowledge) work environment questionnaire that has analyzed category threshold order with Rasch is also the workplace social capital domain of COPSOQ 3. The items in this domain of COPSOQ 3 have five response categories (to a very small extent, to a small extent, somewhat, to a large extent, to a very large extent). The response categories in their items were concentrated in two categories, “somewhat” and “to a large extent”, and the response category “to a very small extent” was rarely used [36], supporting a good category threshold order in the trichotomized scale of the SMET questionnaire.

The trichotomization procedure in the SMET questionnaire is strongly supported due to the minimally affected ability to capture the construct of the work environment, since measurement targeting is in the good/acceptable range and shows good category threshold order. These results are of great importance when evaluating the work environment and the effect of work environment measures.

The Rasch analysis highlights the relevance and usefulness of specific items, which has been seen in other research where reliable and valid questionnaires have been assessed with Rasch analysis [37]. Three items in the SMET questionnaire must be compiled and analyzed with some caution, from a psychometric perspective. Item 7 (Do you experience any problems associated with prolonged sitting in your work?) should be analyzed with care, considering the insufficient category threshold order also in the trichotomized scale. Prolonged sitting is a health hazard which has gained more attention in recent years [38, 39]. It is also a question which may be highly relevant for some professions, while other professions have sitting work duties to a much lesser extent. The SMET questionnaire has been continuously developed and tested in practice [14, 15], where the item has been seen as important, thus item 7 will be kept in the SMET questionnaire despite the category threshold disorder.

Item 22 and item 10 also present challenges to discuss when using the SMET questionnaire in practice, but also when discussing potential future revisions. Item 22 (If you think about your work satisfaction and work environment, how satisfied would you say that you are?) was considered to be an all-embracing item at the start of the development of the SMET questionnaire. However, the factor analysis revealed that this was not the case, and in accordance with previous analysis of the SMET questionnaire it is suggested that in a revision of the SMET questionnaire, item 22 should be omitted and if the current SMET questionnaire is used, this item should be treated with caution when analyzing the results in a specific setting. Item 10 (Do you experience any problems associated with high noise levels at your workplace?) is useful in practical work, according to the practical experience of the OHS researchers in the research group, and therefore it is suggested it should remain in the SMET questionnaire, despite not being among the factors in the trichotomized version. Healthcare localities rarely have high levels of noise as a work environmental issue, which might explain the results. If the SMET questionnaire is used in other contexts, item 10 might be of greater importance [40].

The results of this study present valuable knowledge about the psychometric evaluation of multidisciplinary OHS measurements, a field in need of well-established, valid and reliable measurement tools [2]. The results from this and previous studies support the following use of the SMET questionnaire:

1. The SMET questionnaire should be administered and answered with the initial 1–10 scale. 2. Trichotomization of the 1–10 scale should be conducted before analyzing and presenting the results. 3. The open-ended items are analyzed and compiled with a content analysis, as described in previous research [15].

### 5.2 Practical implications for OHS work and scientific research

The results of this, and our previous studies [14–16] evaluating the psychometric properties of the SMET questionnaire support the use of the SMET questionnaire when evaluating the work environment in healthcare settings. Systematic work environment management (SWEM) is a provision from SWEA that describes mandatory work by the employer to minimize ill-health and accidents at work. SWEM consists of iterative risk assessment, measures, and follow-up as continuous work [41]. The SMET questionnaire might be of importance in practical OHS work in healthcare settings as it provides a psychometrically well-tested method when supporting employers and employees in their SWEM [41]. The SMET questionnaire will help the employer to identify specific work-related problems, since it evaluates the work environment from a broader multifactorial perspective than other measurement methods. The trichotomized results will support the employer in prioritizing their work environment actions. Items identifying work related problems indicates that actions in this area will be of greater importance, since this study shows that the differences in the trichotomized scale (green–yellow–red) are reliable. The good psychometric properties of the SMET questionnaire will also be practically useful to evaluate the results of conducted work environment measures. If there are differences between baseline and follow-up evaluations, these differences will be reliable, showing true changes in the work environment. Supporting employers and employees with SMET in their SWEM might benefit both employers and employees as scientific research has linked work environment conditions to low back pain [42], stress [43] and intention to leave [44], etc. Good working conditions have also been shown to protect nurses from work-related illness during the Covid-19 pandemic [45].

The results might also be of importance in scientific research evaluating the work environment within healthcare. In scientific research the good psychometric properties of the SMET questionnaire are important and will contribute to identification of work environment problems, supporting correct measures to improve the work environment and making it possible to follow up and evaluate the outcome of work environment measures, with high quality, validity and reliability.

Future studies will evaluate the psychometrical properties of the SMET questionnaire in other parts of the labor market.

### 5.3 Strengths and limitations

Due to the lack of psychometrically well-tested methods for evaluation of the work environment in healthcare settings [13] it is a strength of this study that the psychometric evaluation was performed not only with CTT but also with a more sophisticated method like Rasch.

The present Rasch analysis shows that the results of psychometric tests not only provide information regarding the validity and reliability of a questionnaire [46]; they also provide a greater understanding of the psychometric properties of pragmatically useful OHS questionnaires. This is useful for OHS research and practice and will help to further optimize other questionnaires used in the field of occupational health.

The advantage of the Rasch model is its ability to manage anomalies in data from a theoretical point of view. Thus, compared to CTT, considering the results of a Rasch analysis from a qualitative perspective helps researchers to reflect in more sophisticated ways on the constructs (variables) they wish to measure [22]. The use of the Rasch model eliminates certain doubts in the data analysis about weights and equidistance, and allows scores to be summated [47]. All this assumes that the items work invariantly and show proper categorization [48].

The large sample size in this study (*n* = 793) strengthens the value of the results, since sample sizes above *n* = 500 are considered very good in both Rasch analysis [30] and CTT [31].

The absence of item fit to the model analysis in our study might be considered a limitation. Evaluation of item fit to the model is important when item results are added and total scores are summed together for the whole questionnaire or for different questionnaire domains [23]. Item fit analysis was not conducted in this study since all items in the SMET questionnaire are compiled and interpreted separately.

The SMET questionnaire has so far only been tested in healthcare contexts. The psychometric properties of the SMET questionnaire must be evaluated further, if the questionnaire is to be used in other parts of the labor market.

## 6 Conclusion

The results of this study support the use of the SMET questionnaire as a psychometrically well-tested method for a broad multifactorial evaluation of the work environment in healthcare settings. The good psychometric properties of the SMET questionnaire are of great importance and will contribute to identification of work environment problems, supporting correct measures to improve the work environment and making it possible to follow up and evaluate the outcome of work environment measures, with high quality, validity and reliability, both in practical OHS work and scientific research.
